# Understanding Misclassification between Neonatal Deaths and Stillbirths: Empirical Evidence from Malawi

**DOI:** 10.1371/journal.pone.0168743

**Published:** 2016-12-28

**Authors:** Li Liu, Henry D. Kalter, Yue Chu, Narjis Kazmi, Alain K. Koffi, Agbessi Amouzou, Olga Joos, Melinda Munos, Robert E. Black

**Affiliations:** 1 Department of Population Family and Reproductive Health, Johns Hopkins Bloomberg School of Public Health, Baltimore, Maryland, United States of America; 2 The Institute for International Programs, Department of International Health, Johns Hopkins Bloomberg School of Public Health, Baltimore, Maryland, United States of America; 3 Health Systems Program, Department of International Health, Johns Hopkins Bloomberg School of Public Health, Baltimore, Maryland, United States of America; TNO, NETHERLANDS

## Abstract

Improving the counting of stillbirths and neonatal deaths is important to tracking Sustainable Development Goal 3.2 and improving vital statistics in low- and middle-income countries (LMICs). However, the validity of self-reported stillbirths and neonatal deaths in surveys is often threatened by misclassification errors between the two birth outcomes. We assessed the extent and correlates of stillbirths being misclassified as neonatal deaths by comparing two recent and linked population surveys conducted in Malawi, one being a full birth history (FBH) survey, and the other a follow-up verbal/social autopsy (VASA) survey. We found that one-fifth of 365 neonatal deaths identified in the FBH survey were classified as stillbirths in the VASA survey. Neonatal deaths with signs of movements in the last few days before delivery reported were less likely to be misclassified stillbirths (OR = 0.08, p<0.05). Having signs of birth injury was found to be associated with higher odds of misclassification (OR = 6.17, p<0.05). We recommend replicating our study with larger sample size in other settings. Additionally, we recommend conducting validation studies to confirm accuracy and completeness of live births and neonatal deaths reported in household surveys with events reported in a full birth history and the extent of underestimation of neonatal mortality resulting from misclassifications. Questions on fetal movement, signs of life at delivery and improved probing among older mother may be useful to improve accuracy of reported events.

## Introduction

Globally, an estimated 2.6 million stillbirths and 2.7 million neonatal deaths occurred in 2015.[1, 2] Numbers of live births, stillbirths, and neonatal deaths are fundamental vital statistics to inform national and global policy making and resource allocation for newborn survival. Improved counting of stillbirths and neonatal deaths help refine the estimation of live births and other demographic measures, such as neonatal mortality rate, infant mortality rate, under-five mortality rate, and life expectancy, which contribute to the ongoing efforts to improve vital statistics in low- and middle-income countries (LMICs). Better distinction between stillbirths and neonatal deaths could also improve the estimation of causes of stillbirths and neonatal deaths.

In LMICs, full birth history (FBH) and full pregnancy history interviews of women of reproductive age are currently considered the best practice for measuring neonatal mortality. These approaches are widely used by large household survey programs like the Demographic and Health Surveys (DHS) and Multiple Indicator Cluster Surveys (MICS). Such surveys are the primary source of data for pregnancy outcomes and child survival status in LMICs. However, FBH is not always a reliable source for stillbirth and neonatal death estimates.[3, 4] First, stillbirths and neonatal deaths have many causes in common such as asphyxia and prematurity and are sometimes hard to distinguish [5, 6], and sometimes the cause of death classification systems wouldn’t distinguish causes for stillbirths versus neonatal period.[7] Second, babies born with limited signs of life, such as crying, breathing, and movement, who died shortly after birth may be mistakenly or intentionally reported as stillbirths.[8, 9] The concept of perinatal mortality was created because of this well-recognized overlap of stillbirths and neonatal deaths.[10]

The validity of mothers’ reporting of their birth outcomes in surveys is affected by several factors. They include 1) whether mothers possess accurate knowledge to distinguish between stillbirths and live births; 2) whether birth attendants have conducted careful assessments for signs of life; 3) whether birth attendants try to avoid blame, extra work, or audit review; or 4) family or women’s perception of pregnancy losses. [11, 12]

Even if mothers knew at the time of the event whether their babies were stillborn or live born but died soon after, additional factors may affect the validity of their reporting. First, omission in reporting could occur. For example, either stillbirths or neonatal deaths could be too painful for mothers to recall and report.[12] Mothers may choose to conceal them due to social stigma or cultural practices [12] Older women may have more difficulties accurately recalling such events that occurred many years earlier.[13] Survey interviewers could be motivated not to report the event to reduce their workload.[14] Second, misclassification in reporting could occur. Mothers may report neonatal deaths as stillbirths or vice versa either deliberately out of concerns over discrimination or unintentionally due to recall bias.[12] Lastly, linguistic barriers or cultural context could also contribute to the misclassification. Other studies identified several phrases from the local language that could either refer to stillbirths or premature live births.[12, 15] In some African contexts, women bear more blame if their newborns die than if they have had stillbirths. Women may thus intentionally misreport neonatal deaths as stillbirths.[12]

In this study, we aimed to assess and quantify the level of misclassification of stillbirths as neonatal deaths in FBH interviews using data from a large mortality survey carried out in two districts in Malawi followed by verbal/social autopsy (VASA) interviews, treating the latter as the reference standard. We also assessed the correlates of misclassification to provide recommendations on ways to improve FBH interviews to reduce such misclassification.

## Methods

### Data

We analyzed data from two surveys conducted in Malawi—the Real-Time Monitoring of Under-Five Mortality (RMM) FBH survey [16] and the VASA survey.[17] The FBH survey was conducted in Balaka and Salima districts between October 2011 and February 2012.[18, 19] Based on two-stage sampling, 12,000 households in each district were selected with a total sample of 24,000 households. The survey instrument used FBH interviews to measure neonatal mortality among live births. Information on socio-economic characteristics, maternal demographics, and newborn characteristics was also collected from eligible women aged 15–49 years. Survival status and age at death were asked of every reported live birth in the FBH survey with brief probing on vital signs at birth including “ever breathed or cried” or “showed other signs of life” to identify live births in the survey reference period (Table 1). The FBH survey asked only about live births and did not collect data on pregnancy losses (stillbirth, miscarriage, or abortion).

**Table 1:** Questions relevant to the classification between stillbirths and neonatal deaths from the FBH and the VASA surveys.

VASA		FBH	
Question	Answer	Question	Answer
V1.11. Was the child born alive or dead?	1. Alive; 2. Dead; 9. Don’t know	Child Mortality(#CM7): Have you ever given birth to a boy or girl who was born alive but later died? If “No” probe by asking: Any baby who ever breathed or cried or showed other signs of life but did not survive—even if he/or she lived only a few minutes or hours?	YES / No
V1.12. Did the baby ever cry?	1. Yes; 2. No; 9. Don’t know		
V1.13. Did the baby ever move?			
V1.14. Did the baby ever breathe?			
V1.15. Refer to VQ1.11–1.14. If “Dead” & no crying, movement or breathing, mark “Stillbirth.” If “Alive” & VQ1.12–1.14 = “No,” or if “Dead” and VQ1.12, 1.13 or 1.14 = “Yes,” then discuss & correct.	1. Stillbirth; 2. Live birth		
V1.16 and V3.1. Were there any bruises or signs of injury on the baby’s body at birth? (Same for stillbirths and neonatal deaths)	1. Yes; 2. No; 9. Don’t know		
V1.17. (for stillbirths) Was the baby’s body (skin and tissue) pulpy?	1. Yes; 2. No; 9. Don’t know		
V1.18 and V3.2. Was any part of the baby physically abnormal at the time of delivery? (for example: body part too large or too small, additional growth on body) (Same for stillbirths and neonatal deaths)	1. Yes; 2. No; 9. Don’t know		
V1.19 and V3.3. What were the abnormalities? Ask for the following abnormalities: [Mark all that apply—Show photos] (Same for stillbirths and neonatal deaths)	1. Was the head size very small at the time of birth; 2. Was the head size very large at the time of birth; 3. Was there a mass defect on the back of head or spine; 4. Was there any other abnormality (If “Yes,” then specify)		

The VASA survey was conducted in March and April of 2013. The VASA followed up a sample of all neonatal and post neonatal deaths identified by the FBH survey on the 4 years preceding the survey. Only one death was sample per household and VASA interviews were conducted with the mother or immediate caretaker of the deceased child before the death. The VASA interview reassessed whether the deceased children were live born or stillborn.[20] It collected detailed information on signs and symptoms surrounding the birth and on maternal characteristics using a standard VASA questionnaire.[17] Mothers were asked whether the index baby was born alive or not, with probing questions on whether the index baby showed any signs of life, that is whether the baby ever breathed, ever cried, or ever moved. The VASA survey also included several more questions on the appearance of the index baby at birth. Additional questions were also asked on signs of fetal movement prior to the delivery.(Table 1)

Stillbirths were defined in the VASA survey as births reported as born dead and showing no sign of life, that is, no breathing, no movement, and no crying at birth. Cases identified as neonatal deaths in the FBH survey, and as neonatal deaths or stillbirths in the VASA survey were included in the analyses. In total, 476 neonatal deaths identified by the FBH survey as having occurred in the 4 years prior to the FBH survey were sampled by the VASA survey. Of these, 399 had completed interviews. Another 34 cases re-classified as child deaths by the VASA survey were excluded from the analyses. In the end, 365 observations were included in this study.

### Analytical approaches

Because the VASA survey sampled a subset of the FBH survey respondents, we linked records between the two surveys at the individual level, and assessed the extent to which stillbirths had been misclassified as neonatal deaths in the FBH by cross-tabulating the numbers of neonatal deaths and stillbirths identified in both surveys, treating VASA as the reference standard. The misclassification rate was defined as the percent of deaths reported in the FBH survey as neonatal deaths that were later classified as stillbirths in the VASA survey. Given the VASA survey was conducted on neonatal deaths reported through the FBH survey, it was not possible to assess the extent to which true neonatal deaths were omitted from the FBH when respondents incorrectly considered them to be stillbirths. We also cross-tabulated age at death from the two surveys to further assess data quality.

We performed descriptive bivariate analyses between misclassification status and four sets of characteristics, including socio-demographic, babies’, mothers’, and surveys’ characteristics, that could be associated with misclassification. Specifically, socio-demographic characteristics examined included mothers’ education and age, main breadwinner of the household, household wealth quintile (based on a household wealth index created using a series of questions on household assets [21]), and district; babies’ characteristics included whether baby was moving in the last few days before birth, sex of the baby, birth size, birth weight, singleton versus multiple births, birth order in the FBH, place of delivery, place of death, birth injury (indicated by bruises and signs of injury on baby’s body at birth), and congenital abnormalities observed at birth; mothers’ characteristics included number of antenatal care visits, delivery assistance provided by doctor, nurse/midwife, or others, pregnancy duration measured by self-reported gestational age in months and whether preterm versus full-term with a cutoff at nine months, pregnancy complications symptoms, and duration of labor; and surveys’ characteristics included length of recall defined by duration between dates of death and dates of interview in both surveys, and the lag time between dates of interview in the two surveys. Most of these characteristics were collected in the VASA survey. If similar information was collected in both surveys, data from the VASA were used.

We derived means and standard deviations for continuous variables and number and percent of observations for categorical variables. With misclassification as binary outcome variable (yes/no), we conducted bivariate logistic regressions among continuous variables and chi-squared tests among categorical variables to examine bivariate association between misclassification and the characteristics described above.

Variables with a p-value of no more than 0.05 in the bivariate analyses were entered into multivariate logistic regressions with the four sets of characteristics entered sequentially. Specifically babies’ characteristics were entered first in model 1, with maternal and socio-demographic characteristics added in model 2, and survey characteristics further included in model 3. The final model was selected based on likelihood-ratio tests. The analyses were conducted using Stata 14. (Stata/SE 14.1, StataCorp LP) Sensitivity analyses relaxing the inclusion p-value to 0.1 and 0.2 were also conducted.

## Results

Among the 365 cases identified as neonatal deaths in the FBH survey, 75 (20.5%) were classified as stillbirths in the VASA survey (Table 2). Half (50.7%) of the stillbirths were fresh stillbirths, defined as those born with skin still intact.[22]

**Table 2:** Percent of stillbirths misclassified by FBH as neonatal deaths, by district in Malawi.

FBH (neonatal deaths)	VASA			
	Neonatal deaths	Stillbirths	Total	Misclassification (%)
Both districts	290	75	365	20.5
Salima	129	40	169	23.7
Balaka	161	35	196	17.9

**Fig 1:**
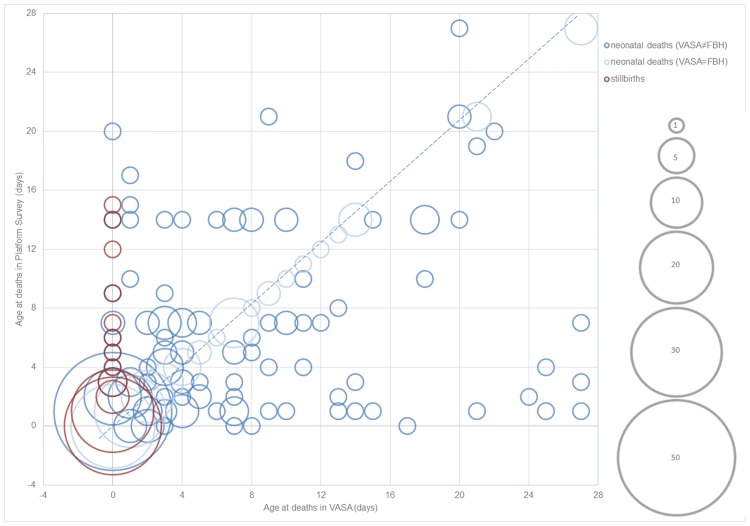
Age at deaths among neonates by survey type, Malawi, 2011–2012*. *Size of the bubbles represents number of deaths, and red bubbles represent cases that were classified as stillbirths in VASA but neonatal deaths in FBH.

Fig 1 shows the comparison of age at death between the FBH and VASA surveys, where size of the bubbles represents the number of deaths, and red bubbles represent misclassified cases. More than 10% (N = 8) of the misclassified cases had a reported age at death ranging between 4 and 15 days in the FBH survey. For the remaining neonatal deaths, the bubbles should line up on the diagonal line if age at death was consistently reported between the two surveys. However, the bubbles scattered all over the figure and some were far-off the diagonal line. Cohen’s kappa coefficient was estimated to be 0.24 (p<0.05), indicating fair agreement on age at death between the two surveys.[23] In addition, age heaping appeared to be an issue on day 7 in both surveys, and on day 14 in the FBH survey.

Socio-demographic, babies’, mothers’, and survey characteristics by misclassification status are presented in Table 3. With regard to socio-demographic characteristics, misclassified cases had older mothers than correctly classified cases (28.01 vs. 26.09 years, p = 0.04). Among babies’ characteristics, misclassified cases were significantly less likely to have reported movement in the last few days before delivery, yet significantly more likely to have reported signs of birth injury (p<0.01). Among mothers’ characteristics, longer reported duration of labor and delivery was associated with misclassification (11.53 vs. 8.10 hours, p = 0.03). None of the surveys’ characteristics was statistically significant.

**Table 3:** Babies’, mothers’, socio-demographic, and survey’s characteristics by FBH misclassification status in Malawi.

Variables	Stillbirth misclassified as neonatal deaths (N = 75)		Correctly classified neonatal deaths (N = 290)		P	Missing
	Mean / N	SD / %	Mean / N	SD / %		
**Socio-demographic characteristics**						
Residence					0.17	0
Salima	40	53.33	129	44.48		
Balaka	35	46.67	161	55.52		
Mother’s age (in years)	28.01	7.69	26.09	7.04	0.04	0
Maternal education					0.59	0
No education	10	13.33	49	16.90		
Primary	57	76.00	203	70.00		
Secondary & Higher	8	10.67	38	13.10		
Marital status					0.91	0
Married/Living with a man	63	84.00	242	83.45		
Single/Widowed/Divorced	12	16.00	48	16.55		
Main Breadwinner					0.64	0
Child’s father	65	86.67	257	88.62		
Child’s mother/other	10	13.33	33	11.38		
Wealth index					0.98	0
Poorest Quintile	20	26.67	77	26.55		
Poor Quintile	11	14.67	47	16.21		
Middle Quintile	14	18.67	56	19.31		
Wealthy Quintile	14	18.67	57	19.66		
Wealthiest Quintile	16	21.33	53	18.28		
**Babies’ characteristics**						
Baby sex						0
Boy	39	52.00	163	56.20	0.51	
Girl	36	48.70	127	43.80		
Single/Multiple Birth						0
Single	68	90.67	252	86.90	0.38	
Multiple	7	9.33	38	13.10		
Birth order					0.26	0
First	22	29.33	98	33.79		
Second	9	12.00	51	17.59		
Third or above	44	58.67	141	48.62		
Baby size at delivery					0.87	27
Very Small/smaller than usual	77	29.23	19	28.20		
About Average/larger than usual	196	70.77	46	71.80		
Reported signs of injury at birth					<0.01	7
No	46	64.79	272	94.77		
Yes	25	35.21	15	5.23		
Baby moving in the last few days before delivery					<0.01	3
No	29	38.67	12	4.18		
Yes	46	61.33	275	95.82		
Place of birth/delivery					0.57	1
On route to a health provider or facility/other	9	12.00	23	7.93		
Hospital	31	41.33	109	37.59		
Other health provider	19	25.33	85	29.31		
Home	16	21.33	73	25.17		
Place of Death					0.18	0
On route to a health provider or facility/other	9	12.00	21	7.24		
Hospital	31	41.33	109	37.59		
Other health provider	19	25.33	50	17.24		
Home	16	21.33	110	37.93		
**Mother’s characteristics**						
Duration of labor and delivery (in hours)	11.53	14	8.10	8.32	0.01	7
Antenatal clinical visits					0.78	7
No visit	4	5.48	17	5.96		
1–3 visits	41	56.16	147	51.58		
at least 4 visits	28	38.36	121	42.46		
Delivery assistance by					0.52	2
Doctor	17	22.67	52	17.93		
Nurse/midwife	31	41.33	139	47.93		
Traditional birth attendant/self/neighbor/relative/other	27	36.00	99	34.14		
Complications during this pregnancy					0.20	0
At least one complication reported*	50	66.67	215	74.14		
No complications during pregnancy	25	33.33	75	25.86		
Pregnancy Duration					0.20	2
Preterm	31	41.33	96	33.33		
Full term	44	58.67	192	66.67		
**Surveys’ characteristics**						
Recall period of VASA (months)	36.88	14.63	38.82	14.25	0.30	0
Recall period of FBH (months)	20.31	14.87	22.46	14.01	0.24	0
Lag between VASA and FBH (months)	16.57	2.16	16.37	1.77	0.39	0

*Complications during pregnancy included: Convulsions; High blood pressure; Severe anemia or pallor or shortness of breath; Diabetes; Severe headache; Blurred vision; Too weak to get out of bed; Severe abdominal pain; Fast or difficult breathing; Puffy face; Any vaginal bleeding before labor; Excessive bleeding during labor or delivery; Fever; Smelly vaginal discharge; Child delivered not head first; Cord delivered first; Cord around the child’s neck; Any other complication reported by respondent.

Multivariate logistic regressions results are shown in Table 4. In the final model, that is model 3, when all characteristics were considered, deaths for which fetal movements was reported before delivery were less likely to be misclassified than those for which such movements were not reported. (OR = 0.08, p<0.05). Deaths with reported signs of birth injury were more likely to be misclassified than those without. (OR = 6.17, P<0.05) In addition, the odds of misclassification increased by 1.04 times for each one-year increase in mothers’ age, but this was only marginally significant (p<0.10). Sensitivity analyses relaxing the inclusion p-value from 0.05 to 0.1 and 0.2 gave similar results.

**Table 4:** Multivariate regression results^&^. (OR: odds ratio; SE: standard error;).

Variables	Model 1 (N = 356)		Model 2 (N = 352)		Model 3 (N = 352)	
	OR	SE	OR	SE	OR	SE
**Babies’ characteristics**						
Baby moved before delivery (yes vs. no)	0.08*	0.04	0.09*	0.04	0.08*	0.04
Birth injury (yes vs. no)	6.33*	2.64	6.15*	2.59	6.17*	2.66
**Mothers’ characteristics**						
Duration of labor (hour)			1.01	0.02	1.01	0.02
**Socio-demographic characteristics**						
Mother’s age (year)					1.04^#^	0.02
Likelihood-ratio Test						
LR Chi2				0.45		3.73
p-value				0.50		0.05

^&^Outcome is a binary variable indicating whether the case was a misclassified stillbirth (yes/no).

*p<0.05;

^#^p<0.10

## Discussion

In this study, we assessed the extent and correlates of stillbirths being misclassified as neonatal deaths by comparing two linked population surveys, a FBH survey and a VASA survey in Malawi. Treating the VASA survey as the reference standard, we found that overall one-fifth of neonatal deaths identified in the FBH were stillbirths as classified by the VASA survey. Deaths without reported fetal movement right before birth, with reported birth injury and with older mothers were found more likely to be misclassified.

Previous studies have found that mothers’ perception of reduced fetal movements is associated with stillbirth. [24, 25] This is consistent with our finding that stillborn babies, though misclassified as neonatal deaths in the FBH survey, were less likely to move in the last few days before birth. For cases with birth injury, we speculate that it could be hard to determine clinically whether they were live births or stillbirths, and hence the higher probability of misclassification. The association between older age and misclassification may be interpreted as increased recall biases associated with aging.[26]

Our study did not have a large sample size, so the findings should be interpreted with caution. We suggest that similar but larger studies should be conducted in other contexts to assess the robustness of these findings. Constrained by the sample size, we were not able to further investigate whether the association between misclassification and the examined characteristics differ by fresh versus other stillbirths. Qualitative studies with the mothers and/or interviewers could be suggested to further look into the causes of misclassification, especially the ones misclassified non-fresh stillbirths as neonatal deaths. Only information on live births were collected in the FBH survey. As a result, we could only identify one-way misclassification, that is stillbirths misclassified as neonatal deaths; yet misclassification could happen the other way as well.[11] Future research could be done examining full pregnancy history and validate the reported pregnancy outcomes to explore the misclassification both ways, and to evaluate the impact of misclassification on neonatal mortality and child mortality estimates.

We also found it intriguing that the agreement in the reporting of age at death was only fair between the two surveys. Potential data quality limitations such as respondent or interviewer error could be present in both the FBH and VASA surveys with few cases providing contradictory information regarding the dates of events. Though striking, this may not be entirely unexpected. One contributor to the inconsistency could be age heaping. [11] A previous study found that Africa has the highest level of age displacement compared to other regions partially due to lower female school attainment.[27] However, with an 87% female literacy rate in Malawi,[28] education level may not be an important barrier for correct reporting of age at death.

77 of the neonatal deaths identified in the FBH weren’t able to complete the VASA interview, mainly due to vacant, destroyed or not found households, especially in Salima where had higher mobility of the fishing households that migrate frequently depending on fishing season. We examined the main characteristics such as age at deaths, mothers’ age, maternal education, recall period of the surveys etc. based on the information from FBH survey and found no difference between those with complete VASA and those lost-to-follow-up (with degree of significance at 0.05). We suspect that the bias introduced by loss-to-follow-up would be minimal in our analysis.

Treating VASA as the reference standard is a strong assumption. We hypothesized that more information on stillbirths collected in the VASA surveys than in the FBH survey, specifically on whether babies’ ever cried, moved or breathed, may help improve classification validity. The mechanisms could be that additional verbal autopsy questions helped women better recall. However, we were not able to test our assumption or hypothesis, and we acknowledge that classification in the VASA survey may not be accurate either. As a result, the extent of misclassification could be either under- or over-estimated.

In general, it seems challenging to add additional questions to the already lengthy FBH instrument used in DHS or Multiple Indicator Cluster Surveys.[14] But if adding more questions is feasible, additional individual questions on ever cried, ever moved, or ever breathed should be considered. In addition, collecting information on fetal movements right before births and birth injury may be helpful to improve reporting validity of stillbirths and neonatal deaths. Our findings also suggest that additional and perhaps repeated probing among older women might be helpful to improve their reporting validity.

These additional questions can be tested in stand-alone formative research or as additional questions in surveys including FBH, full pregnancy history, or VASA. The advantage of testing these questions using a VASA survey is the thorough questioning of the event that may solicit detailed information. Therefore, we recommend validation studies to determine the validity of these questions, including those on ever crying, breathing and moving. Validation studies can be conducted in places such as health facilities, home deliveries attended by trained midwives, and health and demographic surveillance sites where better reference standards of stillbirths and neonatal deaths are available. Cognitive testing could also be done to assess the feasibility of adding the questions to FBH surveys. If the questions demonstrate validity and feasibility, they can be incorporated into other survey instruments. Studies could also be done to test the utility of the open history section in VASA surveys in reducing misclassification. Currently the open history section has very limited responses. Additional emphasis during training on its use, and the inclusion of leading statements, such as “Was your baby moving a few days before birth?” could be included to ensure that more useful information is collected in the open history section.

Improving the counting of stillbirths and neonatal deaths is not only important to the tracking of global development goals [29] and the monitoring of national development status, but also relevant to the continued efforts on improving vital statistics in LMICs.[2, 30] We provided some recommendations for future research in this area, which hopefully could contribute to this important global health and development agenda.
